# Extracellular phospholipase production by *Malassezia pachydermatis* strains and its inhibition by selected antimycotics and plant essential oil components

**DOI:** 10.1007/s11259-024-10446-5

**Published:** 2024-06-26

**Authors:** Eva Čonková, Peter Váczi, Zuzana Malinovská

**Affiliations:** Department of Pharmacology and Toxicology, University of Veterinary Medicine and Pharmacy in Košice, Komenského 73, Košice, Slovakia

**Keywords:** Azole antimycotics, Plant essential oil components, *Malassezia pachydermatis*, Extracellular phospholipase activity

## Abstract

Extracellular phospholipase (EPL) plays an important role in the pathogenesis of the yeast *Malassezia pachydermatis*. Currently, the attention of researchers is focused on studying the virulence factors involved in this process and searching solutions to reduce their activity. One of the options is the use of natural remedies as anti-virulence agents. This study is aimed at investigating the production of extracellular phospholipase in *M. pachydermatis* strains (18 samples) and followed by the time-dependent inhibitory effect of selected azole antifungals (itraconazole, posaconazole and voriconazole) and plant essential oil components (terpinen-4-ol, thymol, carvacrol, eugenol and geraniol), evaluated by Egg Yolk Agar plate method. Almost all strains (17 isolates, (94.4%) were found to be intense EPL producers. A significant, time-dependent inhibition of EPL was noted after 1-, 3- and 6-h exposure of *Malassezia* cells to itraconazole (26.4%, 47.2% and 50.9%, respectively) compared to exposure to posaconazole (26.4%, 28.3% and 28.3%, respectively) and voriconazole (18.8%, 20.8% and 35.8%, respectively). After one-hour exposure to plant essential oil components, the best inhibitory effect was recorded for eugenol (62.3%), followed by terpinen-4-ol and thymol (56.6%), geraniol (41.5%) and carvacrol (26.4%). A 3-h exposure revealed that thymol retained the best inhibitory effect (88.7%) on EPL production, followed by carvacrol (73.6%), eugenol (56.6%), terpinen-4-ol (52.8%) and geraniol (49.1%). After 6-h exposure, no growth of *M. pachydermatis* strains exposed to carvacrol was observed, and the inhibitory efficiency for the other tested essential oil (EO) components achieved 88.7%. The obtained results indicate the promising efficacy of plant essential oils components in the inhibition of virulence factors such as EPL production.

## Introduction

The genus *Malassezia*, an opportunistic yeast, represents an important part of the microbiome of healthy skin and mucous membranes of humans, warm-blooded animals and birds (Puig et al. 2017; Theelen et al. 2018). Currently, 18 species are included in this genus (Lorch et al. 2018; Hobi et al. 2022), of which *Malassezia (M.) pachydermatis* plays a significant role in canine diseases presented as *Malassezia* dermatitis or otitis (Bond et al. 2020; Bajwa 2023). *Malassezia* occurs predominantly in dogs with long hair and pendulous ears (Čonková et al. 2011) and with excessive sebum production and/or decreased quality of sebum (seborrhoea), accumulation of moisture, damage of epidermis and concomitant dermatoses, atopy, and bacterial skin infections (Patterson and Frank 2002; Ugochukwu et al. 2023). The yeast can become pathogenic when the physical, chemical or immunological mechanisms of the skin are altered (Buommino et al. 2016). The switch from a commensal microorganism to a pathogen is influenced by several factors, including the ability to produce enzymes such as esterases, lipases, lipoxygenases, and proteases that promote yeast growth on the host´s skin (Celis et al. 2017). The production of phospholipase, one of the important virulence factors, was also confirmed in *M. pachydermatis* isolated in dogs with or without skin lesions and with or without otitis (Cafarchia and Otranto 2004; Ortiz et al. 2013). Phospholipases (PLs) are a heterogeneous group of enzymes that catalyse the hydrolysis of glycerophospholipids by cleaving one or more ester bonds, resulting in the release of free fatty acids that trigger inflammation in the host (Tee et al. 2019; Talapko et al. 2021).

Treatment of *Malassezia* infections in dogs and cats is based on the use of preparations containing polyene antimycotics (nystatin) or azoles such as clotrimazole, itraconazole, fluconazole, posaconazole, and miconazole. There are no information whether commonly used antifungal drugs also affect the production of extracellular phospholipase in *M. pachydermatis* strains. Due to limitation effect of antifungals and the increased development of resistance to them, researchers are looking for alternative methods of treatment. The components of plant essential oils represent a group that worth to explore not only for their antifungal properties but also for their effect on some virulence factors, such as the production of extracellular phospholipase by *Malassezia* strains.

The main goal of this study was to evaluate the inhibitory effect of selected components of essential oils (terpinen-4-ol, thymol, carvacrol, eugenol and geraniol) on the production of extracellular phospholipase in *M. pachydermatis* strains and to compare their effect with three azole antimycotics (itraconazole, posaconazole and voriconazole).

## Material and methods

### *Malassezia pachydermatis* strains

Eighteen *M. pachydermatis* strains isolated from auricular swabs of dogs without current manifestation and recent history of otitis externa were used for the testing. There was no known history of antifungal treatment in the preceding 3 months. The samples were taken from dogs of different breeds, sexes and ages, patients of the Small Animal Clinic of the University of Veterinary Medicine and Pharmacy in Košice, Slovakia. All strains used for the experiments were identified and confirmed based on their phenotypic and genotypic characteristics described by Kaneko et al. (2007) and Gaitanis et al. (2002). Until the beginning of the experiments, the strains were kept at -80 °C in a freezing medium (100 µL 60% glycerol and 300 µL medium – glucose 4 g, tryptophan 1 g, yeast extract 0.5 g per 100 mL). Before usage, the yeasts were revived twice on SAOT (Sabouraud’s dextrose agar – SDA—HiMedia Laboratories Pvt. Ltd, Mumbai India, supplemented with glycerol – 2 mL, Tween 80 – 2 mL, Tween 40 – 5 mL and olive oil – 5 mL per litre) and incubated at 35 °C for 96 h. The reference strain of *M. pachydermatis* CBS 1879 (Centraalbureau voor Schimmelcultures, Utrecht, The Netherlands) was also included in the study.

### Determination of extracellular phospholipase activity (Pz index)

Extracellular phospholipase production was assessed using the Egg Yolk Agar plate method described by Jain et al. (2017), with small modifications. Briefly, a few colonies were picked from each *M. pachydermatis* isolate cultured on SAOT and transferred to approximately 5 mL of sterile Phosphate-buffered saline (PBS) solution with 0.1% Tween 80. Using a densitometer (Pliva-LaChema a.s., Brno, Czech Republic), the cell suspension was adjusted to an optical density at McFarland 1 which corresponds to approximately 1–5 × 10^6^ CFU/mL. An aliquot of 10 µL yeast suspension was spot inoculated onto the surface of the Egg Yolk Agar medium (65 g SDA, 58.4 g NaCl, and 5.5 g CaCl_2_ per litre, sterilised at 121 °C for 15 min; 100 mL of Egg Yolk emulsion 50%—HiMedia Laboratories Pvt. Ltd, Mumbai India, was added to the medium cooled at 45–50 °C) and allowed to dry at room temperature. After this, the plates were incubated at 35 °C for 10 days. Each isolate was tested in duplicate. The value of phospholipase activity (Pz) was determined by the ratio of the diameter of the colony alone to the diameter of the colony with the precipitation zone. The Pz value was classified into five categories as follows: Pz = 1 (negative); 0.90–0.99 (weak); 0.89–0.80 (poor); 0.79–0.70 (moderate); ≤ 0.69 (intense) (Fule et al. 2015).

### Determination of minimal inhibitory concentration (MIC) of tested antifungals and plant essential oil components

For this purpose, the standard method M27-A3 (CLSI 2008) was used with some modifications. The antifungal activity of three antifungal agents: itraconazole, posaconazole and voriconazole (Sigma Aldrich, St. Louis, USA), and five components of plant essential oils: terpinen-4-ol, thymol, carvacrol, eugenol and geraniol (Sigma Aldrich, St. Louis, USA) were tested against *M. pachydermatis* strains.

First, a stock solution (1,600 µg/mL) of antimycotics tested was prepared by dissolving them with dimethyl sulfoxide (DMSO). Then, the solutions were diluted with SBOT medium (Sabouraud’s broth medium supplemented with the same substances as SAOT) to a concentration of 32 µg/mL.

In the case of plant essential oil components, 100% concentration of the plant EO component was dissolved in 40% DMSO to a concentration of 50% (500 mg/mL) and consequently using SBOT to the required stock solution concentration of 25 mg/mL (25,000 µg/mL).

In both cases, the final DMSO concentration reached 2% and the solutions were used to prepare the tested concentrations by binary dilution directly in the microplates. Wells 1–10 contained either the concentrations of antimycotics in the range of 32.0–0.0625 µg/mL or EO components in the range of 25 – 0.1 mg/mL (25,000–100 µg/mL) in an amount of 100 µL. Two hundred microlitres of SBOT was added into the well number 11 (negative control). Then, 100 µL of the *Malassezia* suspension containing 10^4^ CFU/mL (prepared by diluting 10^6^ CFU/mL suspension with SBOT at a ratio of 1:100) was added into wells 1–10 and 12 (positive control). An additional 100 µL of SBOT was added into well 12 to keep the volume in each well the same. Adding the *Malassezia* suspension into the wells halved the concentrations of antimycotics to 16 – 0.0313 µg/mL and of EO components to 12,500 – 50 µg/mL. The microplates were then incubated at 35 °C for 72 h and then the minimal inhibitory concentration (MIC) was read. To better evaluate the MIC end-points, a colorimetric method was used, by adding 10 µL of 0.1% resazurin (sterilised through 0.22 µm filter before use) into each well of microplate six hours before reading the results. Inhibition of yeast growth was determined at the MIC that prevented the change from blue (no yeast growth) to orange-pink (yeast growth) (Liu et al. 2007).

### Testing the reduction of phospholipase production by antifungals and plant essential oil components

For this assay, the method described by Ellepola et al. (2016) was adopted, with some modifications. Two millilitres of the inoculum suspension (10^6^ CFU/mL) and 2 mL of a fourfold MIC concentration of antifungals or plant EO component were added to the tubes, so that the final concentration of each agent tested was twice the MIC concentrations. The cells were exposed to these agents for 1, 3, and 6 h at a temperature of 35 °C. The control tube from each isolate contained only 2 mL of inoculum suspension without the tested antifungal agents. After the exposure period, the test agents were removed by undergoing two wash cycles with 5 mL of PBS and centrifugation (10 min at 3,000 rpm). Then, 2 mL of PBS were added to the sedimented cells, and mixed well and 10 µL of each isolate (control and drug-exposed) was applied onto the Egg Yolk Agar surface. The plates were incubated at 35 °C for 10 days. After this time the activity of EPL (Pz) was assessed as described above. Based on the Pz value, the percentage inhibition of EPL activity was calculated by the following equation:

$$\mathrm{Inhibition}\;\left(\%\right)=\lbrack1-(\text{Pz sample}/\text{Pz control})\rbrack\times100.$$

### Statistical analysis

Each experiment was repeated twice and average values were taken. The data are presented as average means ($\overline{\mathrm x}$), standard deviations (SD), mode and median. One-way ANOVA followed by Tukey’s multiple comparisons test was used to compare the mean MICs of the selected antifungal agents with each other and also to compare the mean Pz (EPL activity) of exposed cells with unexposed cells after 1-, 3-, and 6-h exposure (GraphPad Prism 8.0.1, San Diego, CA, USA). The level of statistical significance was set up at *p* < 0.05.

## Results

The statistical evaluation of the MIC (µg/mL) of the tested antifungal drugs found in *M. pachydermatis* isolates and the reference strain is presented in Table 1. Itraconazole with the average MIC 0.97 µg/mL showed the lowest (*p* < 0.05) antifungal efficacy compared to posaconazole with a mean MIC of 0.43 µg/mL, and voriconazole with a mean MIC of 0.19 µg/mL. In *M. pachydermatis* CBS 1879 strain, the highest antifungal activity was found for voriconazole (MIC = 0.125 µg/mL), followed by posaconazole (MIC = 0.25 µg/mL) and itraconazole (MIC = 0.5 µg/mL).

**Table 1:** Evaluation of MIC (µg/mL) of tested antifungals

Parameter	Itraconazole	Posaconazole	Voriconazole
*Malassezia pachydermatis* isolates			
Range	0.25–4	0.25–1	0.125–0.25
$\overline{\mathrm x}$	0.97^a,b^	0.43^a^	0.19^b^
SD	1.12	0.19	0.06
Mode	0.5	0.5	0.25
Median	0.5	0.25	0.19
MIC_50_	0.5	0.25	0.125
MIC_90_	1	0.5	0.25
*Malassezia pachydermatis* CBS 1879			
Range	0.5	0.25	0.125
$\overline{\mathrm x}$	0.5	0.25	0.125
SD	0	0	0
Mode	0.5	0.25	0.125
Median	0.5	0.25	0.125

$\overline{\mathrm x}$ mean, *SD* standard deviation, *MIC* minimum inhibitory concentration of antifungal agent that prevents visible growth of a microorganism, *MIC*_*50*_*/MIC*_*90*_ minimum inhibitory concentration at which the growth is inhibited in 50/90% isolates, a-b – MIC mean values with the same superscript letter are statistically significantly different (*p* < 0.05) as analysed by one-way ANOVA and Tukey’s test

Table 2 represents the statistical analysis of MIC values of the tested plant EO components used against *M. pachydermatis* strains. Eugenol (average MIC = 344.44 µg/mL) was found to have the best antifungal efficacy (*p*˃0.05), followed by thymol (mean MIC = 773.89 µg/mL), carvacrol (mean MIC = 1,195.56 µg/mL), terpinen-4-ol (mean MIC = 1,216.11 µg/mL) and geraniol (mean MIC = 1,522.78 µg/mL), respectively. The *M. pachydermatis* reference strain also showed the best susceptibility to eugenol (800 µg/mL), followed by terpine-4-ol (1,600 µg/mL) and thymol (1,600 µg/mL).

**Table 2:** Evaluation of MIC (µg/mL) in tested component of plant essential oils

Parameter	Terpinen-4-ol	Thymol	Carvacrol	Eugenol	Geraniol
*Malassezia pachydermatis* isolates					
Range	100–3,130	100–3,130	100–3,130	50–1,600	50–3,130
$\overline{\mathrm x}$	1,216.11^a^	773.89	1,195.56^b^	344.44^a,b,c^	1,522.78^c^
SD	1,000.59	862.85	1,215.57	431.10	1,368.21
Mode	800	1,600	100	100	3,130
Median	800	400	800	100	800
MIC_50_	800	400	800	100	800
MIC_90_	3,130	1,600	3,130	800	3,130
*Malassezia pachydermatis* CBS 1879					
Range	1,600	1,600	3,130	800	3,130
$\overline{\mathrm x}$	1,600	1,600	3,130	800	3,130
SD	0	0	0	0	0
Mode	1,600	1,600	3,130	800	3,130
Median	1,600	1,600	3,130	800	3,130

$\overline{\mathrm x}$ mean, *SD* standard deviation, *MIC* minimum inhibitory concentration of tested palnt essential oil component that prevents visible growth of a microorganism, *MIC*_*50*_*/MIC*_*90*_ minimum inhibitory concentration at which the growth is inhibited in 50/90% isolates, ^a–c ^– MIC mean values with the same superscript letter are statistically significantly different (*p* < 0.05) as analysed by one-way ANOVA and Tukey’s test

The basic statistics of the Pz index means calculated from the two measurements is shown in Table 3.

**Table 3:** Evaluation of phospholipase activity (means of Pz index) of *M. pachydermatis* isolates and reference strain CBS 1879

*M. pachydermatis*	Range	$\overline{\mathrm x}$±SD	Mode	Median	Negative *n*/%	Intense *n*/%
Isolated strains (*n* = 18)	0.40–1	0.55 ± 0.13	0.50	0.55	1/5.6	17/94.4
Reference strain (CBS 1879)	0.67	0.67 ± 0	0.67	0.67	–	1/100

$\overline{\mathrm x}$ mean, *SD* standard deviation, *n* number of strains

The mean of the Pz values (0.55 ± 0.13 for the isolates and 0.67 ± 0 for the reference strain) indicate high EPL activity in the tested strains. Out of the 18 M*. pachydermatis* strain isolates, only one strain (5.6%) showed no EPL activity, while 17 strains (94.4%) were assessed with intense EPL activity. The reference strain, *M. pachydermatis* CBS 1879, tested in triplicate, showed intense EPL activity.

**Fig. 1:**
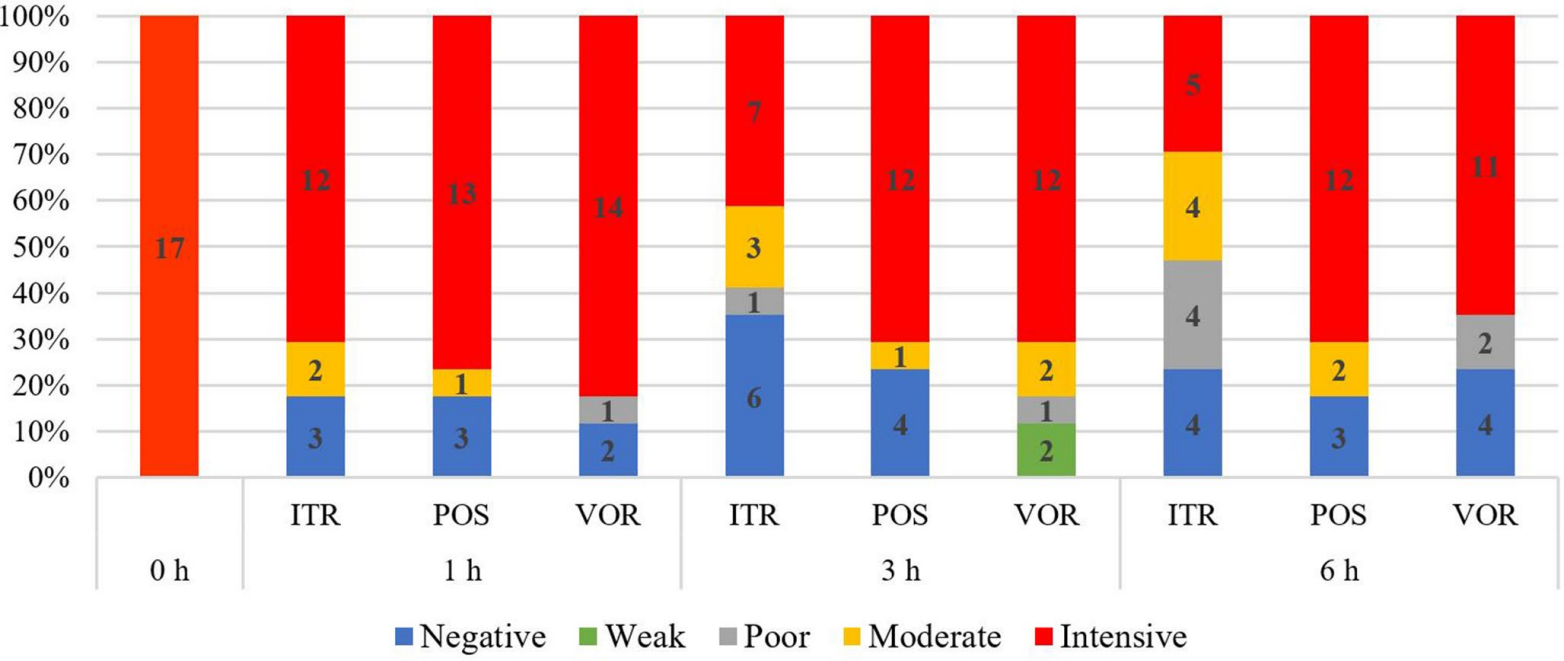
The intensity of phospholipase activity of *M. pachydermatis* isolates before and after exposure to itraconazole, posaconazole and voriconazole for 1 h, 3 h and 6 h

Statistical analyses of phospholipase activity and the percentage of inhibitory efficiency of the tested antimycotics on phospholipase activity, depending on the time of exposure, is demonstrated in Table 4. A significant reduction (*p* < 0.05) in EPL activity was noted after 1-, 3-, and 6-h exposure to itraconazole with inhibitory efficiencies of 26.4%, 47.2%, and 50.9%, respectively, compared to unexposed cells. For posaconazole, the percentage of inhibitory efficacy on EPL activity reached 26.4% after 1-h exposure, 28.3% after 3-h and 6-h exposure. An increasing, time-dependent inhibitory effect was also observed for voriconazole with 18.9% after 1-h exposure, 20.8% after 3-h exposure and significant inhibition of 35.8% after 6-h exposure (*p* < 0.05). No statistical differences were found in the inhibition of EPL production depending on the time for individual tested antifungal drugs. In the case of the reference strain, *M. pachydermatis* CBS 1879, the same inhibitory effect (49.3%) was recorded for all tested antimycotics. These results are consistent with the data presented in Table 5 and depicted in Fig. 1, showing the changes in EPL activity after exposure to antimycotics.

**Table 4:** Statistical evaluation of phospholipase activity (Pz index) of *M. pachydermatis* strains before and after exposure to itraconazole, posaconazole and voriconazole for 1 h, 3 h and 6 h and inhibition effectivity (%)

Parameter	Unexposed	Itraconazole			Posaconazole			Voriconazole		
		1 h	3 h	6 h	1 h	3 h	6 h	1 h	3 h	6 h
*Malassezia pachydermatis* isolates										
Range	0.4–0.67	0.47–1	0.46–1	0.55–1	0.47–1	0.49–1	0.53–1	0.50–1	0.45–1	0.45–1
$\overline{\mathrm x}$	0.53^a,b,c,d,e,f,g^	0.67^a^	0.78^b^	0.80^c^	0.67^d^	0.68^e^	0.68^f^	0.63	0.64	0.72^ g^
SD	0.06	0.17	0.19	0.14	0.18	0.19	0.16	0.16	0.14	0.18
Mode	0.50	0.57	1	1	0.67	0.57	1	0.50	0.56	1
Median	0.54	0.60	0.77	0.78	0.63	0.57	0.62	0.57	0.60	0.67
Inhibition (%)	–	26.4	47.2	50.9	26.4	28.3	28.3	18.9	20.8	35.8
*Malassezia pachydermatis* CBS 1879										
Range	0.67	1	1	1	1	1	1	1	1	1
$\overline{\mathrm x}$	0.67	1	1	1	1	1	1	1	1	1
SD	0	0	0	0	0	0	0	0	0	0
Mode	0.67	1	1	1	1	1	1	1	1	1
Median	0.67	1	1	1	1	1	1	1	1	1
Inhibition (%)	–	49.3	49.3	49.3	49.3	49.3	49.3	49.3	49.3	49.3

$\overline{\mathrm x}$ mean, *SD* standard deviation; ^a–f ^– Pz mean values with the same with the same superscript letter are statistically significantly different (*p* < 0.05) as analysed by one-way ANOVA and Tukey’s test

**Table 5:** Evaluation of phospholipase activity of *M. pachydermatis* isolates before and after exposure to itraconazole, posaconazole and voriconazole for 1 h, 3 h and 6 h based on Pz index

EPL activity	Unexposed	Itraconazole			Posaconazole			Voriconazole		
		1 h (*n*/%)	3 h (*n*/%)	6 h (*n*/%)	1 h (*n*/%)	3 h (*n*/%)	6 h (*n*/%)	1 h (*n*/%)	3 h (*n*/%)	6 h (*n*/%)
Negative	–	3/17.6	6/35.3	4/23.5	3/17.6	4/23.5	3/17.6	2/11.8	–	4/23.5
Weak	–	–	–	–	–	–	–	–	2/11.8	–
Poor	–	–	1/5.9	4/23.5	–	–	–	1/5.9	1/5.9	2/11.8
Moderate	–	2/11.8	3/17.6	4/23.5	1/5.9	1/5.9	2/11.8	–	2/11.8	–
Intense	17/100	12/70.6	7/41.2	5/29.5	13/76.5	12/70.6	12/70.6	14/82.3	12/70.5	11/64.7

*n* number of strains

Compared to the 17 unexposed strains with intensive EPL production (100%), after 1-h exposure to the tested antifungals, the intense EPL activity decreased and reached 12 strains (70.6%) for itraconazole, 13 isolates (76.5%) for posaconazole, and 14 strains (82.3%) for voriconazole. After a 3-h exposure to itraconazole, 7 strains (41.2%) were found to have intense EPL activity, however, for posaconazole and voriconazole, EPL activity declined only slightly, to 12 isolates (70.6%) for both. Similarly, after 6-h exposure, the highest decrease of intense EPL activity was recorded with itraconazole– 5 strains (29.5%), compared to posaconazole– 12 strains (70.6%), and voriconazole– 11 isolates (64.7%).

A statistically significant difference (*p* < 0.05) in the inhibition of EPL activity was observed at almost all exposure times for the isolates of *M. pachydermatis* exposed to plant EO components when comparing with EPL production of unexposed cells (Table 6). Among the tested plant EO components, eugenol (62.3%) had the highest inhibitory effect on EPL activity of *M. pachydermatis* after 1-h exposure, followed by terpinen-4-ol and thymol (56.6%), geraniol (41.5%) and carvacrol (26.4%). It was found that thymol (88.7%) retained the best inhibitory effect on the EPL activity of *Malassezia* yeasts even after a 3-h exposure, followed by carvacrol (73.6%), eugenol (56.6%), terpinen-4-ol (52.8%) and geraniol (49.1%). After 6-h exposure, no growth of *M. pachydermatis* strains exposed to carvacrol was observed, and the inhibitory efficiency for the other tested EO components achieved 88.7%. For the *M. pachydermatis* CBS 1879 strain, when comparing unexposed cells and cells exposed to EO components, an inhibitory effect on EPL activity of 14.9% and 43.9% was recorded for terpinen-4-ol and geraniol, respectively, after 1-h exposure. After a 3-h exposure, inhibition of EPL activity was detected only for terpinen-4-ol (49.3%). For the other EO components tested, no growth of the *M. pachydermatis* reference strain was observed at other times.

**Table 6:** Statistical evaluation of phospholipase activity (Pz index) of *M. pachydermatis* isolates before and after exposure to terpinen-4-ol, thymol, carvacrol, eugenol and geraniol for 1 h, 3 h and 6 h, and inhibition effectivity (%)

Parameter	Unexposed	Terpinen-4-ol			Thymol			Carvacrol			Eugenol			Geraniol		
		1 h	3 h	6 h	1 h	3 h	6 h	1 h	3 h	6 h	1 h	3 h	6 h	1 h	3 h	6 h
*Malassezia pachydermatis* isolates																
Range	0.4–0.67	0.50–1	0.56–1	1	0.54–1	1	1	0.50–1	0.77–1	–	0.54–1	0.62–1	1	0.52–1	0.60–1	1
$\overline{\mathrm x}$	0.53^a,b,c,d,e,f,g,h,i,j,k,l,m^	0.83^a^	0.81^b^	1^c^	0.83^d^	1^e^	1^f^	0.67	0.92^ g^	–	0.86^ h^	0.83^i^	1^j^	0.75^ k^	0.79^ l^	1^ m^
SD	0.06	0.20	0.18	0	0.20	0	0	0.23	0.13	–	0.17	0.15	0	0.19	0.20	0
Mode	0.50	1	1	1	1	1	-	1	1	–	1	1	1	1	1	1
Median	0.54	1	0.79	1	0.92	1	1	0,6	1	–	0.92	0.76	1	0.72	0.67	1
Inhibition (%)	–	56.6	52.8	88.7	56.6	88.7	88.7	26.4	73.6	–	62.3	56.6	88.7	41.5	49.1	88.7
*Malassezia pachydermatis* CBS 1879																
Range	0.67	0.71–0.83	1	–	–	–	–	–	–	–	–	–	–	1	–	–
$\overline{\mathrm x}$	0.67	0.77	1	–	–	–	–	–	–	–	–	–	–	1	–	–
SD	0	0.06	0	–	–	–	–	–	–	–	–	–	–	–	–	–
Mode	0.67	–	1	–	–	–	–	–	–	–	–	–	–	–	–	–
Median	0.67	0.77	1	–	–	–	–	–	–	–	–	–	–	1	–	–
Inhibition (%)	–	14.9	49.3	–	–	–	–	–	–	–	–	–	–	49.3	–	–

$\overline{\mathrm x}$ mean, *SD* standard deviation; ^a–m ^– Pz mean values with the same superscript letter are statistically significantly different (*p* < 0.05) as analysed by one-way ANOVA and Tukey’s test

**Fig. 2:**
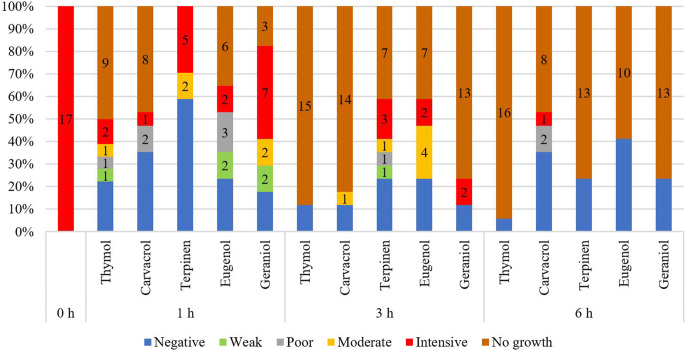
The intensity of phospholipase activity of *M. pachydermatis* isolates before and after exposure to EO components for 1 h, 3 h and 6 h

When comparing changes in the intensity of EPL activity of *M. pachydermatis* isolates (Table 7 and Fig. 2), the highest inhibitory effect was found in strains exposed to thymol for 1 h, as only two isolates (11.8%) showed intense EPL activity and the growth of 10 strains (58.8%) was completely inhibited. This phenomenon was also recorded after a 3-h exposure to thymol, when 15 strains (88.2%) of *Malassezia* yeast showed no growth and in two samples (11.8%) no EPL activity was detected. No EPL production was noted even after 6-h exposure when one isolate revealed negative EPL activity (5.9%) and 16 isolates (94.1%) showed no yeast growth. Carvacrol, as another of the tested EO components, was found to have high inhibitory efficiency. Increased time-dependent inhibition of yeast growth of was recorded after 1-, 3- and 6-h exposure, 47.1%, 82.3% and 100%, respectively. Only 2 isolates (11.8%) with intense EPL activity were noticed after 1-h and 3-h exposure to eugenol, while no growth of yeasts was registered at the same time in 6 strains (35.3%) and in 7 strains (41.2%), respectively. After 6 h of exposure to eugenol, no yeast EPL activity was observed, as 7 isolates (41.2%) were not EPL producers and 10 strains did not grow. For terpinen-4-ol, intense EPL activity was reduced to 5 strains (29.4%), after a 1-h exposure. Strains exposed to terpinen-4-ol for 3-h showed intense EPL activity of 17.6% (3 isolates) and no growth of 41.2% (7 strains). No EPL activity of yeasts was detected in strains exposed for 6 h. *M. pachydermatis* strains exposed to geraniol indicated the EPL activity in 7 isolates (41.2%) after 1-h exposure, and in 3 strains (17.6%) after 3-h exposure. Similar to other EO components tested, no EPL production was noticed after a 6-h exposure to geraniol.

**Table 7:** Evaluation of the intensity of phospholipase activity of unexposed *M. pachydermatis* isolates and after exposure to terpinene-4-ol, thymol, carvacrol, eugenol and geraniol for 1 h, 3 h and 6 h based on Pz index

Parameter	Unexposed	Terpinen-4-ol			Thymol			Carvacrol			Eugenol			Geraniol		
		1 h (*n*/%)	3 h (*n*/%)	6 h (*n*/%)	1 h (*n*/%)	3 h (*n*/%)	6 h (*n*/%)	1 h (*n*/%)	3 h (*n*/%)	6 h (*n*/%)	1 h (*n*/%)	3 h (*n*/%)	6 h (*n*/%)	1 h (*n*/%)	3 h (*n*/%)	6 h (*n*/%)
*Malassezia pachydermatis* isolates																
Negative	–	9/53.0	4/23.5	4/23.5	3/17.6	2/11.8	1/5.9	4/23.5	2/11.8	–	4/23.5	4/23.5	7/41.2	3/17.6	2/11.8	4/23.5
Weak	–	–	–	–	1/5.9	–	–	–	–	–	2/11.8	–	–	2/11.8	–	–
Poor	–	–	1/5.9	–	–	–	–	–	–	–	3/17.6	–	–	-	–	–
Moderate	–	3/17.6	2/11.8	–	1/5.9	–	–	–	1/5.9	–	–	4/23.5	–	2/11.8	–	–
Intense	17/100	5/29.4	3/17.6	–	2/11.8	–	–	5/29.4	–	–	2/11.8	2/11.8	–	7/41.2	3/17.6	–
No colony growth	–	–	7/41.2	13/76.5	10/58.8	15/88.2	16//94.1	8/47.1	14/82.3	17/100	6/35.3	7/41.2	10/58.8	3/17.6	12/70.6	13/76.5
*Malassezia pachydermatis* CBS 1879																
Negative	–	–	3/100	–	–	–	–	–	–	–	–	–	–	1/33.3	–	–
Weak	–	–	–	–	–	–	–	–	–	–	–	–	–	–	–	–
Poor	–	1/33.3	–	–	–	–	–	–	–	–	–	–	–	–	–	–
Moderate	–	2/66.7	–	–	–	–	–	–	–	–	–	–	–	–	–	–
Intense	3/100	–	–	–	–	–	–	–	–	–	–	–	–	–	–	–
No colony growth	–	–	–	3/100	3/100	3/100	3/100	3/100	3/100	3/100	3/100	3/100	3/100	2/66.7	3/100	3/100

## Discussion

Current treatment of *Malassezia* infection in animals is based on the use of common antifungals, especially azoles. Itraconazole and posaconazole, triazole derivatives, are most often preferred for the treatment. While itraconazole is used orally, posaconazole, a second-generation triazole antifungal agent derived from the structure of itraconazole, was introduced as an aural topical formulation (Peano et al. 2020; Ding et al. 2022). Voriconazole, a second-generation triazole that is derived from fluconazole, is not currently available in any drug formulation for veterinary use, but the susceptibility of *M. pachydermatis* to this agent was determined in a study by Cafarchia et al. (2015), where its MIC range reached 0.016– 0.5 µg/mL in strains isolated from dogs with localized or generalized dermatitis. MIC values of itraconazole (0.008–0.125 µg/mL) and posaconazole (0.008–0.032 µg/mL) are also reported in this study. However, compared to the results of the cited authors, our isolates revealed generally higher MIC values. We assume that the reason for hte higher MIC values in this study may be determined mainly by the use of different test conditions or due to higher natural resistance of the collected strains. Similar to infections caused by *Candida* species, researchers are focusing on the virulence attributes responsible for the development of pathogenicity also in the *Malassezia* diseases (Angiolella et al. 2023). It has been found that lipolytic enzymes including extracellular phospholipase may contribute to the virulence of pathogenic fungi such as *Candida albicans*, *Cryptococcus neoformans* and *Aspergillus fumigatus*. The phospholipase activities were detected also in *M. furfur*, *M. pachydermatis*, *M. slooffiae, M. sympodialis*, *M. globosa*, *M. restricta* and *M. obtusa* (Juntachai et al. 2009). There is evidence that phospholipases may contribute to host cell penetration, injury, and lysis (Park et al. 2013; Singh et al. 2023). In the study of Cafarchia and Otranto (2004), a significant phospholipase activity (93.9%, mean Pz value = 0.72) was reported by *M. pachydermatis* strains collected from dogs with lesions compared to the isolates obtained from healthy skin sites of the same dog with localized lesions (41.8%, mean Pz value = 0.89) or isolates collected from skin sites of healthy dogs (10.6%, Pz value = 0.97). Similarly, the highest intensity of phospholipase activity (29.5%) was found in *M. pachydermatis* isolates acquired from dogs with otitis compared to dogs without otitis (13.7%) (Ortiz et al. 2013). Out of the 18 strains of *M. pachydermatis* included in our study, only one strain (5.6%) showed no EPL activity and the rest (94.4%) were intense EPL producers. In our opinion, these results are in accordance with the data of the susceptibility of the strains to the tested antifungal agents, for which higher MIC values were found. Since EPL activity represents an important virulence factor, this study also looked at the possibilities of its inhibition. The obtained results show that the inhibition of EPL production increased time-dependently after exposure to itraconazole from 26.4% to 50.9%, to posaconazole from 26.4% to 28.3%, and to voriconazole from 18.9% to 35.8%. Close to our research, but with different antifungal agents against EPL produced by *C. albicans* isolates, has been carried out so far only by Ellepola et al. (2016), so we could not compare the results obtained with any other authors.

In recent years, interest in the treatment of *M. pachydermatis* infections with natural plant products has considerably raised due to the growing resistance to antifungals (Bismarck et al. 2020; Ebani and Mancianti 2020). Investigation of new antifungal agents for their impact on virulence is an important tool for exploring novel antifungal targets leading to improved therapeutic regimens. Plant essential oils and their active compounds deserve attention in this regard (El-Baz et al. 2021). Several articles have dealt with the antifungal effect of plant essential oils against *M. pachydermatis* (Khosravi et al. 2016; Váczi et al. 2018), but there are scanty data on the antifungal efficacy of their components, nor on their inhibitory activity against *M. pachydermatis* virulence factors, such as EPL production. In general, plant EOs are complex mixtures of natural compounds, and are well-known for their antiseptic and medicinal properties (analgesic, sedative, anti-inflammatory, spasmolytic, local anaesthetic, anti-carcinogenic, antibacterial, antifungal and antiviral) (Raut and Karuppayil 2014; Nazzaro et al. 2017). Most essential oils are composed of terpenes, terpenoids, and other aromatic and aliphatic constituents with low molecular weights. Few articles on *M. pachydermatis*, point to the antifungal or antibiofilm effect of EO components such as thymol, carvacrol and eugenol (Aiemsaard et al. 2019; Schlemmer et al. 2019; Sim et al. 2019), but none on terpinen-4-ol.

In the present article, the antifungal activity of the above-mentioned EO components was assayed, as well as their inhibitory effect on EPL production by *M. pachydermatis* strains. All of the tested EO components induced good antifungal efficacy and were able to reduce EPL production. When evaluating the antifungal activity of the tested plant essential oil components, eugenol was found to be the most effective (mean of MIC = 344.44 µg/mL). Its inhibitory effect on EPL activity reached 62.3% after 1-h exposure and 56.6% and 88.7% after 3 and 6-h exposure, respectively. Eugenol is an aromatic compound belonging to the group of monoterpenoid phenols. It is commonly obtained from the natural essential oils of plants from the *Lamiaceae*, *Lauraceae*, *Myrtaceae* and *Myristicaceae* families, and is the most important component of clove oil (*Syzygium aromaticum*), where it constitutes between 9,381.7 mg and 14,650 mg per 100 g of fresh plant material and is primarily responsible for its characteristic aroma (Ulanowska and Olas 2021). Growth inhibition of *M. pachydermatis* planktonic cells by eugenol was detected at the MIC_50_ of 0.156 mg/mL, and its fungicidal effect at the minimum fungicidal concentration (MFC) of 0.312 mg/mL. Thymol was another compound that exhibited excellent results in inhibiting the growth of *M. pachydermatis* (mean of MIC = 773.89 µg/mL) and EPL production in the range of 56.6%—88.7% after 1–6 h of exposure. It is a natural volatile monoterpenoid phenol, which is the main active component of the oil extracted from species *Thymus vulgaris* L., and other plants such as *Ocimum gratissimum* L., *Origanum vulgare* L., various species of the genus *Satureja* L., (*Lamiaceae*), *Carum copticum* L. and *Oliveria decumbens* Vent (*Apiaceae*), and many others (Escobar et al. 2020). Carvacrol, a monoterpene phenol is the main constituent of EO extracted from oregano belonging to the *Lamiaceae* family (Memar et al. 2017). In our study, higher antifungal activity against *M. pachydermatis* isolates was found for thymol compared to carvacrol (mean of MIC = 1,195.56 µg/mL), in contrast to the findings of Sim et al. (2019), where the isolates were more sensitive to carvacrol, with the MIC_90_ of 585 µg/mL, as for thymol with the MIC90 of 800 µg/mL. Although the EPL inhibition by carvacrol reached only 26.4% after 1-h exposure, an increasing effect (73.6%) was showed after 3-h exposure and complete inhibition was noted after 6-h exposure, as no colony growth of tested strains was found. Terpinen-4-ol, a monoterpene compound, also showed good antifungal efficacy (mean of MIC = 1,216.11 µg/mL) and inhibitory activity against EPL after 1-, 3- and 6-h (56.6%, 52.8% and 88.7%, respectively) exposure. Terpinen-4-ol is one of the major constituents of tea tree oil (representing at least 30% of the oil) that is extracted from the plant *Melaleuca alternifolia* Cheel, belonging to the botanical family of *Myrtaceae* (Francisconi et al. 2020). Weseler et al. (2002) reported the susceptibility of *M. pachydermatis* strains to tea tree oil containing 40.7% terpinen-4-ol in the MIC range of 560–1,120 µg/mL. In this study, geraniol had the lowest antifungal activity (mean of MIC 1,522 µg/mL), and inhibited the EPL activity by 41.5%, 49.1% and 88.7% after 1-, 3- and 6-h exposure, respectively. Geraniol is an acyclic isoprenoid monoterpene alcohol, extracted from the essential oils of *Pelargonium graveolens*, *P. roseum*, *P. odoratissimum* (*Geraniaceae*) and several other plants (Szutt et al. 2020). It is also found in some essential oils such as ninde oil (66.0%), rose oil (44.4%), palmarosa oil (53.5%) and citronella oil (24.8%) (Chen and Viljoen 2010). The potential antifungal activity of geraniol has been demonstrated against dermatophytes, *Aspergillus* species and *C. albicans* strains (Madan and Devaki, 2015).

When comparing the results of evaluating the intensity of EPL activity of *M. pachydermatis* strains, a significant difference is evident. Among the three antifungals tested, itraconazole was the most effective, as the intense EPL activity decreased to 29.5% after 6 h exposure, while at that same time it reached 70.6% for posaconazole and 64.7% for voriconazole. However, after 1-h and 3-h exposure to the tested EO components, the intensity of EPL production decreased rapidly, and no growth of yeast colonies was observed after 6-h exposure. In fungi, one of the main targets of antifungal molecules is cell membrane and the biosynthesis of ergosterol, which is important for the stability of the fungal cell membrane, and inhibition of its biosynthesis compromises cell membrane integrity. Triazole drugs such as itraconazole, posaconazole, and voriconazole block ergosterol synthesis by inhibiting the activity of one of the important enzyme, lanosterol 14α-demethylase, the CYP51-dependent enzyme, which is essential for the conversion of lanosterol to ergosterol. In most of the azoles, the accumulation of lanosterol leads to a fungistatic effect in yeast and a fungicidal effect in moulds, except at high doses, while voriconazole and itraconazole are considered to have fungicidal inhibition (Scott and Simpson 2007; Shafiei et al. 2020; Ding et al. 2022). The mode of action of EOs or their main components is studied more on *Candida* species. Ergosterol synthesis is also the target of most plant EOs and their major components, leading to disruption of the cell membrane and cell wall and affecting cell growth and morphology (Nazzaro et al. 2017). De Castro et al. (2015) found a good antifungal activity of thymol on *Candida* spp. related to its binding to ergosterol in the membrane, leading to a decrease in membrane permeability. Carvacrol, an isomer of thymol, is thought to act by disrupting and depolarizing the plasma membrane by targeting membrane proteins and intracellular drug targets. A study in *C. albicans* shows that carvacrol could be a stressor of the endoplasmic reticulum (ER) because the ER of carvacrol-treated cells is fragmented, leading to a disruption of ER organization and to the unfolded protein response by activation of genes involved in proteolysis, amino acid metabolism, phospholipid translocation, oxidative stress response and DNA repair mechanism, and by inhibition of genes involved in ribosome biogenesis, glycosylation, sugar transport, drug export and nuclear import. This mechanism of action may differ from that of thymol, although the molecules are similar (D’agostino et al. 2019). The interaction of eugenol with *C. albicans* examined by electron microscopy showed multiple sites of action in *C. albicans*, such as injuries to cytoplasmic contents, cell membranes, and cell walls after treatment with 200 μl/ml eugenol. Exposure of *C. albicans* cells to eugenol resulted in 50% cell death and a 76% reduction in ergosterol biosynthesis. Therefore, the ability of eugenol to bind to the *C. albicans* membrane and reduce ergosterol biosynthesis is associated with the cell wall and cell membrane damage (Didehdar et al. 2022). Tea tree oil (containing terpinen-4-ol) has been found to have an effect on biological membranes, damaging their integrity and inhibiting the action of enzymes incorporated to increase membrane fluidity with subsequent leakage of intracellular components. Chemical constituents are characteristically hydrophobic and will accumulate in the lipid-rich environments of cell membrane structures and cause structural and functional damage (Francisconi et al. 2020). Unlike the EO components described above, ergosterol has not been found to be a direct target of geraniol. Geraniol is thought to exert an inhibitory effect on the calcineurin pathway, resulting in damage to the plasma membrane and cell wall (D’agostino et al. 2019). Based on this information, we believe that prolonged exposure of *M. pachydermatis* strains to the tested EO components led to cell damage which was manifested by no growth of colonies.

The results acquired in this study confirm the potential possibility of using plant EO components for their antifungal activity and suppression of the factors responsible for the virulence of the yeast *M. pachydermatis*. However, to implement the investigated natural products as additive remedies in veterinary clinical practice, it is necessary to perform in vivo experiments.

## Data Availability

No datasets were generated or analysed during the current study.

## References

[CR1] Aiemsaard J, Kamollerd C, Uopasai S, Singh R, Thongkham E (2019) Efficiency of clove essential oil against planktonic cells and biofilms of *Malassezia pachydermatis* isolated from canine dermatitis. Thai J Vet Med 49(4):415–420. https://he01.tci-thaijo.org/index.php/tjvm/article/view/241553/

[CR2] Angiolella L, Rojas F, Mussin J, Giusiano G (2023) Modulatory effect of *Origanum vulgare* essential oil and carvacrol on *Malassezia* spp. virulence factors. https://pubmed.ncbi.nlm.nih.gov/36958865 10.1093/mmy/myad026 36958865

[CR3] Bajwa J (2023) *Malassezia* species and its significance in canine skin disease. Can Vet J 64(1):87–90. https://www.ncbi.nlm.nih.gov/pmc/articles/PMC9754143 PMC9754143 36593939

[CR4] Bismarck D, Dusold A, Heusinger A, Müller E (2020) Antifungal in vitro activity of essential oils against clinical isolates of *Malassezia pachydermatis* from Canine ears: a report from a practice laboratory. Complement Med Res 27(3):143–154. https://pubmed.ncbi.nlm.nih.gov/31775141 10.1159/000504316 PMC7384348 31775141

[CR5] Bond R, Morris DO, Guillot J, Bensignor EJ, Robson D, Mason KV, Kano R, Hill PB (2020) Biology, diagnosis and treatment of *Malassezia* dermatitis in dogs and cats: clinical consensus guidelines of the world association for veterinary dermatology. Vet Dermatol 31(1):27-e4. https://pubmed.ncbi.nlm.nih.gov/31957204/10.1111/vde.12834 31957203

[CR6] Buommino E, Nocera FP, Parisi A, Rizzo A, Donnarumma G, Mallardo K, Fiorito F, Baroni A, De Martino L (2016) Correlation between genetic variability and virulence factors in clinical strains of *Malassezia pachydermatis* of animal origin. New Microbiol 39(3):216–23. https://pubmed.ncbi.nlm.nih.gov/27284984/27284984

[CR7] Cafarchia C, Otranto D (2004) Association between phospholipase production by *Malassezia pachydermatis* and skin lesions. J Clin Microbiol 42(10):4868–4869. 10.1128/jcm.42.10.4868-4869.2004/15472366 10.1128/JCM.42.10.4868-4869.2004 PMC522356

[CR8] Cafarchia C, Iatta R, Immediato D, Puttilli MR, Otranto D (2015) Azole susceptibility of *Malassezia pachydermatis* and *Malassezia furfur* and tentative epidemiological cut-off values. Med Mycol 53:743–748. 10.1093/mmy/myv049/26162472 10.1093/mmy/myv049

[CR9] Celis Ramirez AM, Wösten HAB, Triana S, Restrepo S, de Cock H (2017) *Malassezia* spp. beyond the mycobiota. SM Dermatolog J 3(3):1019. 10.36876/smdj.1019

[CR10] CLSI - Clinical and Laboratory Standard Institute (2008) Reference method for broth dilution antifungal susceptibility testing of yeasts approved standard, M27-A3 guideline, 3rd edn. CLSI, p 25

[CR11] Chen W, Viljoen AM (2010) Geraniol - A review of a commercially important fragrance material. S Afr J Bot 76(4):643–651. 10.1016/j.sajb.2010.05.008

[CR12] Čonková E, Sesztáková E, Páleník Ľ, Smrčo P, Bílek J (2011) Prevalence of *Malassezia pachydermatis* in dogs with suspected *Malassezia* dermatitis or otitis in Slovakia. Acta Vet Brno 80(3):249–254. 10.2754/avb201180030249

[CR13] D’agostino M, Tesse N, Frippiat JP, Machouart M, Debourgogne A (2019) Essential oils and their natural active compounds presenting antifungal properties. Molecules 24(20):3713. 10.3390/molecules24203713 31619024 10.3390/molecules24203713 PMC6832927

[CR14] de Castro RD, de Souza TM, Bezerra LM, Ferreira GL, Costa EM, Cavalcanti AL (2015) Antifungal activity and mode of action of thymol and its synergism with nystatin against *Candida* species involved with infections in the oral cavity: an in vitro study. BMC Complement Altern Med 15:417. 10.1186/s12906-015-0947-2. https://pubmed.ncbi.nlm.nih.gov/26601661 10.1186/s12906-015-0947-2 PMC4659158 26601661

[CR15] Didehdar M, Chegini Z, Shariati A (2022) Eugenol: A novel therapeutic agent for the inhibition of *Candida* species infection. Front Pharmacol 13:872127. 10.3389/fphar.2022.872127 36016558 10.3389/fphar.2022.872127 PMC9395595

[CR16] Ding Q, Huang S, Sun Z, Chen K, Li X, Pei Q (2022) A review of population pharmacokinetic models of posaconazole. Drug Des Devel Ther 3691–709. 10.2147/DDDT.S384637 10.2147/DDDT.S384637 PMC9584355 36277600

[CR17] Ebani VV, Mancianti F (2020) Use of essential oils in veterinary medicine to combat bacterial and fungal infections. Vet Sci 7(4):193. 10.3390/vetsci7040193 33266079 10.3390/vetsci7040193 PMC7712454

[CR18] El-Baz AM, Mosbah RA, Goda RM, Mansour B, Sultana T, Dahms TES, El-Ganiny AM (2021) Back to nature: combating *Candida **albicans* biofilm, phospholipase and hemolysin using plant essential oils. Antibiotics 10(1):81. 10.3390/antibiotics10010081 33467766 10.3390/antibiotics10010081 PMC7830859

[CR19] Ellepola AN, Samaranayake LP, Khan ZU (2016) Extracellular phospholipase production of oral *Candida albicans* isolates from smokers, diabetics, asthmatics, denture wearers and healthy individuals following brief exposure to polyene, echinocandin and azole antimycotics. Brazil J Microbiol 47:911–916. 10.1016/j.bjm.2016.06.009 10.1016/j.bjm.2016.06.009 PMC5052368 27522928

[CR20] Escobar A, Perez M, Romanelli G, Blustein G (2020) Thymol bioactivity: a review focusing on practical applications. Arab J Chem 13(12):9243–9269. 10.1016/j.arabjc.2020.11.009

[CR21] Francisconi RS, Huacho PMM, Tonon CC, Bordini EAF, Correia MF, Sardi JCO, Spolidorio DMP (2020) Antibiofilm efficacy of tea tree oil and of its main component terpinen-4-ol against *Candida albicans*. Braz Oral Res 34:e050. 10.1590/1807-3107bor-2020.vol34.0050 32578760 10.1590/1807-3107bor-2020.vol34.0050

[CR22] Fule SR, Das D, Fule RP (2015) Detection of phospholipase activity of *Candida albicans* and non albicans isolated from women of reproductive age with vulvovaginal candidiasis in rural area. Ind J Med Microbiol 33(1):92–95. 10.4103/0255-0857.148392 10.4103/0255-0857.148392 25560009

[CR23] Gaitanis G, Velegraki A, Frangoulis E, Mitroussia A, Tsigonia A, Tzimogianni A, Katsambas A, Legakis NJ (2002) Identification of *Malassezia* species from patient skin scales by PCR-RFLP. Clin Microbiol Infect 8:162–173. 10.1046/j.1469-0691.2002.00383.x 12010171 10.1046/j.1469-0691.2002.00383.x

[CR24] Hobi S, Cafarchia C, Romano V, Barrs VR (2022) *Malassezia*: zoonotic implications, parallels and differences in colonization and disease in humans and animals. J Fungi (basel) 8(7):708. 10.3390/jof8070708 35887463 10.3390/jof8070708 PMC9324274

[CR25] Jain C, Das S, Saha R, Ramachandran VG, Bhattacharya SN, Dar S (2017) Detection of phospholipase production by egg yolk-agar in *Malassezia* isolates from diseased and healthy human host. Int J Med Sci Public Health 6(5):1–5. 10.5455/ijmsph.2017.1267623012017

[CR26] Juntachai W, Oura T, Murayama SY, Kajiwara S (2009) The lipolytic enzymes activities of *Malassezia* species. Med Mycol 47(5):477–484. 10.1080/13693780802314825 18798119 10.1080/13693780802314825

[CR27] Kaneko T, Makimura K, Abe M, Shiota R, Nakamura Y, Kano R, Hasegawa A, Sugita T, Shibuya S, Watanabe S, Yamaguchi H, Abe S (2007) Okamura NRevised culture-based system for identification of *Malassezia* species. J Clin Microbiol 45(11):3737–3742. 10.1128/jcm.01243-07/17881545 10.1128/JCM.01243-07 PMC2168522

[CR28] Khosravi AR, Shokri H, Fahimirad S (2016) Efficacy of medicinal essential oils against pathogenic *Malassezia* sp. isolates. J Mycol Med 26(1):28–34. 10.1016/j.mycmed.2015.10.012 10.1016/j.mycmed.2015.10.012 26597143

[CR29] Liu M, Seidel V, Katerere DR, Gray AI (2007) Colorimetric broth microdilution method for the antifungal screening of plant extracts against yeasts. Methods 42:325–329. 10.1016/j.ymeth.2007.02.013 17560320 10.1016/j.ymeth.2007.02.013

[CR30] Lorch JM, Palmer JM, Vanderwolf K J, Schmidt K Z, Verant ML, Weller TJ, Blehert DS (2018) Persoonia - *Malassezia vespertilionis* sp. *nov.:* a new cold-tolerant species of yeast isolated from bats. Mol Phylo Evol Fungi 41:56–70. 10.3767/persoonia.2018.41.04 10.3767/persoonia.2018.41.04 PMC6344816 30728599

[CR31] Madan KA, Devaki T (2015) Geraniol, a component of plant essential oils-a review of its pharmacological activities. Int J Pharm Pharm Sci 7:2013–2016. https://journals.innovareacademics.in/index.php/ijpps/article/view/5271/8613/

[CR32] Memar MY, Raei P, Alizadeh N, Aghdam MA, Kafil HS (2017) Carvacrol and thymol: strong antimicrobial agents against resistant isolates. Rev Res Med Microbiol 28(2):63–68. 10.1097/MRM.0000000000000100

[CR33] Nazzaro F, Fratianni F, Coppola R, Feo V (2017) Essential oils and antifungal activity. Pharmaceuticals (basel) 10(4):86. 10.3390/ph10040086 29099084 10.3390/ph10040086 PMC5748643

[CR34] Ortiz G, Martín MC, Carrillo-Muñoz AJ, Payá MJ (2013) Phospholipase and proteinase production by *Malassezia pachydermatis* isolated in dogs with and without otitis. Rev Iberoam Micol 30(4):235–238. 10.1016/j.riam.2013.01.006 23428748 10.1016/j.riam.2013.01.006

[CR35] Park M, Do E, Jung WH (2013) Lipolytic enzymes involved in the virulence of human pathogenic fungi. Mycobiology 41(2):67–72. 10.5941/MYCO.2013.41.2.67 23874127 10.5941/MYCO.2013.41.2.67 PMC3714442

[CR36] Patterson AP, Frank LA (2002) How to diagnose and treat *Malassezia* dermatitis in dogs. Vet Med 97:612–22. https://api.semanticscholar.org/CorpusID:209306203

[CR37] Peano A, Johnson E, Chiavassa E, Tizzani P, Guillot J, Pasquetti M (2020) Antifungal resistance regarding *Malassezia pachydermatis*: where are we now? J Fungi 6(2):93. 10.3390/jof6020093 10.3390/jof6020093 PMC7345795 32630397

[CR38] Puig L, Bragulat MR, Castellá G (2017) Cabañes FJ Characterization of the species *Malassezia pachydermatis* and re-evaluation of its lipid dependence using a synthetic agar medium. PLoS ONE 12(6):e0179148. 10.1371/journal.pone.0179148 28586389 10.1371/journal.pone.0179148 PMC5460872

[CR39] Raut JS, Karuppayil SM (2014) A status review on the medicinal properties of essential oils. Ind Crop Prod 62:250–264. 10.1016/j.indcrop.2014.05.055

[CR40] Scott LJ, Simpson D (2007) Voriconazole: a review of its use in the management of invasive fungal infections. Drugs 67(2):269–298. 10.2165/00003495-200767020-00009 17284090 10.2165/00003495-200767020-00009

[CR41] Schlemmer KB, Jesus FPK, Tondolo JSM, Weiblen C, Azevedo MI, Machado VS, Botton SA, Alves SH, Santurio JM (2019) In vitro activity of carvacrol, cinnamaldehyde and thymol combined with antifungals against *Malassezia pachydermatis*. J Mycol Med 29(4):375–377. 10.1016/j.mycmed.2019.08.003 31455580 10.1016/j.mycmed.2019.08.003

[CR42] Shafiei M, Peyton L, Hashemzadeh M, Foroumadi A (2020) History of the development of antifungal azoles: a review on structures, SAR, and mechanism of action. Bioorg Chem 104:104240. 10.1016/j.bioorg.2020.104240 32906036 10.1016/j.bioorg.2020.104240

[CR43] Sim JXF, Khazandi M, Chan WY, Trott DJ, Deo P (2019) Antimicrobial activity of thyme oil, oregano oil, thymol and carvacrol against sensitive and resistant microbial isolates from dogs with otitis externa. Vet Dermatol 30(6):524–e159. https://pubmed.ncbi.nlm.nih.gov/31566822/10.1111/vde.12794 31566822

[CR44] Singh V, Rai R, Mathew BJ, Chourasia R, Singh AK, Kumar A, Chaurasiya SK (2023) Phospholipase C: underrated players in microbial infections. Front Cell Infect Microbiol 13:1089374. 10.3389/fcimb.2023.1089374 37139494 10.3389/fcimb.2023.1089374 PMC10149971

[CR45] Szutt A, Dołhańczuk-Śródka A, Sporek M (2020) Evaluation of chemical composition of essential oils derived from different species leaves. Ecol Chem Eng S 26(4):807–816. 10.1515/eces-2019-0057

[CR46] Talapko J, Juzbašić M, Matijević T, Pustijanac E, Bekić S, Kotris I, Škrlec I (2021) *Candida albicans* - the virulence factors and clinical manifestations of infection. J Fungi 7(2):79. 10.3390/jof7020079 10.3390/jof7020079 PMC7912069 33499276

[CR47] Tee CB, Sei Y, Kajiwara S (2019) Secreted hydrolytic and haemolytic activities of *Malassezia* clinical strains. Mycopathologia 184:227–238. 10.1007/s11046-019-00330-1 30919309 10.1007/s11046-019-00330-1

[CR48] Theelen B, Cafarchia C, Gaitanis G, Bassukas ID, Boekhout T, Dawson TL Jr (2018) *Malassezia* ecology, pathophysiology, and treatment. Med Mycol 56(suppl_1):S10–S25. 10.1093/mmy/myx134. Erratum in: Med Mycol. 2019; 57 (3):e2 10.1093/mmy/myx134 29538738

[CR49] Ulanowska M, Olas B (2021) Biological properties and prospects for the application of eugenol - a review. Int J Mol Sci 22(7):3671. 10.3390/ijms22073671 33916044 10.3390/ijms22073671 PMC8036490

[CR50] Ugochukwu ICI, Rhimi W, Chebil W, Rizzo A, Tempesta M, Giusiano G, Tábora RFM, Otranto D, Cafarchia C (2023) Part 2: understanding the role of *Malassezia* spp. in skin disorders: pathogenesis of *Malassezia* associated skin infections. Expert Rev Anti Infect Ther. 10.1080/14787210.2023.2274500 10.1080/14787210.2023.2274500 37883035

[CR51] Váczi P, Čonková E, Marcinčáková D, Sihelská Z (2018) Antifungal effect of selected essential oils on *Malassezia pachydermatis* growth. Folia Vet 62(2):67–72. 10.2478/fv-2018-0018

[CR52] Weseler A, Geiss HK, Saller R, Reichling J (2002) Antifungal effect of Australian tea tree oil on *Malassezia pachydermatis* isolated from canines suffering from cutaneous skin disease. Schweiz Arch Tierheilkd 144(5):215–221. 10.1024/0036-7281.144.5.215 12070905 10.1024/0036-7281.144.5.215

